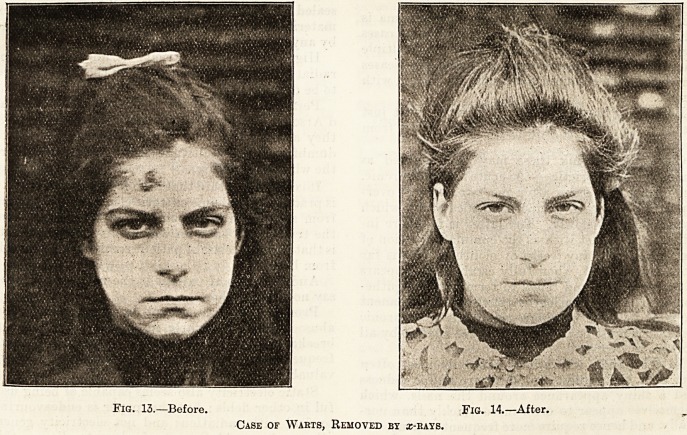# The Present Position of Radiation in Treatment

**Published:** 1906-06-16

**Authors:** Gerald Sichel

**Affiliations:** Surgeon-in-charge of the Actino-therapeutic Department, Guy's Hospital.


### Hospital Clinics.

### THE PRESENT POSITION OF RADIATION IN TREATMENT.

By Gerald Sichel, F.R.C.S., F.C.S., Surgeon-in-charge of the Actino-therapeutic Department, Guy's Hospital.

### III.—*X*-Rays.

There are two great difficulties accompanying treatment with the *x*-rays: the first is that, besides *x*-rays, *x*-ray tubes give out so many other sorts of rays; the second is that, up to the present, we have no means of exactly measuring the amount of rays given out.

We can measure very exactly by means of a delicate measuring instrument (the milliamperemeter) the amount of current which goes into a tube; but there we stop.

There are two factors which have to be determined—(1) the penetration and (2) the intensity of the rays, and in all the methods suggested up to the present must be added the personal equation of both the maker and the operator. Hence the difficulty of exact dosage.

Individual workers, knowing their own particular apparatus, and relying on their own particular form of measurement, can work with success and safety; but at the present time nothing can definitely be said as to dosage generally, except that it is arbitrary, and each man has set up unto himself a separate god.

Until this arbitrary dosage becomes replaced by a scientifically determined, universally recognised, and easily applied measurement, *x*-ray therapy, although an extremely valuable remedy, must remain as a safe measure in the hands of the few only who have conscientiously made a particular study of their own particular apparatus, and will be liable to the adverse influence of the many, who, trading on occasional marvellous results, plunge recklesly into the unknown for purely commercial reasons.

This is why I say it is desirable that a central institute should be established for radio-therapy, which, although only some ten or fifteen years old, has already proved to the hilt its power for both good and evil.

I think that one of the most important recent advances has been the introduction of a "current rectifier" or the more simple "spark gap" in the secondary circuit, to altogether do away with the undesirable "make" current. Certainly this has utterly eliminated one very pregnant cause of uncertainty in tubes.

Another important fact now recognised is that the frequency of interruptions in the primary circuit decidedly controls the amount of current in the secondary circuit, and hence the amount of *x*-rays generated in a tube. Advantage has been taken of this fact by Dr. MacLeod, of Charing Cross Hospital, and he has designed a most ingenious apparatus, which is in use also at the London Hospital, for the treatment of ringworm. When this instrument is used, the whole apparatus is first standardised, and then the treatment is ordered in terms of interruptions—such as 10,000 interruptions, or so forth, as the dose required for a particular case.

Such an arrangement necessarily requires very stable tubes, and stable tubes are not easy to lay hands on.

Recently I have been working with a tube made by Gaiffe, of Paris: this is worked with the anode at red heat, and was claimed to be particularly reliable and steady. I find, however, that in constant working it is not so, and requires constant attention. The same may be said for the so-called self-regulating tubes.

*X*-ray tube makers, such as Messrs. Cossor, Cox, etc., have made enormous strides in the construction of tubes, but the tube which will work at the same tension for eight or ten hours at a stretch is not yet obtainable; at least, I have not been able to come across it in my somewhat extensive experience during the past three years.

Ringworm is one of the diseases which, it is at present claimed, is most successfully treated with *x*-rays, and it is the disease in which most accurate dosage is needed. The desideratum to be arrived at is depilation from a single sitting.

Now depilation means a certain amount of reaction or dermatitis. If the dose is insufficient the hair remains, and the treatment is a failure; and if the dose is too much, not only the hair comes out, but superficial ulceration, with destruction of the hair follicles and permanent baldness results, which may not spell failure so far as the ringworm is concerned, but means disaster for both patient and surgeon.

It stands to reason that very reliable and exact methods of measurement are hence required.

Adamson, whose experience makes his opinion a very valuable one, considers that Sabouraud's pastilles (if the proper make be obtained) are a very safe guide; Batten relies on a galvanoscope; Dore on the appearance of the tube and length of spark-gap. Other workers are guided simply by experience gained by careful observation and use of their particular apparatus.

In my hands Sabouraud's pastilles, I must confess, although I have carefully observed all the precautions necessary, have not given reliable results.

Personally I have treated—chiefly from Sir Cooper Perry's out-patients, which he has sent to me—about 150 cases. The hospital out-patient class is notoriously an extremely difficult one to base statistics on, but, so far as I can make out, my successes with $x$-ray treatment in ringworm are 50 per cent., with an incidence of the disaster of permanent baldness of under 1 per cent.

of cures in a large department comprising all classes is the most that can be expected from $x$-rays in the treatment of ringworm.

Fig. 9.—Before.

Fig. 10.—After.

Case of Ulcerating Lupus treated with $x$-rays: the resulting Scars are much Disfigured by Tetangiectasis.

Fig. 11.—Before.

Fig. 12.—After.

Case of Rodent Ulcer treated by $x$-rays.

Other $x$-ray specialists claim far better results than this, and it is not for me to criticise their figures. My own impression, whether right or wrong, is that for the average worker 50 per cent.

This means a cure within four months, and there is not, and never has been, any other treatment that would give such a good result as this.

I think under present circumstances—and in spite of Sabouraud's cases—that the present-day method of attempting to bring about a cure in one long sitting is too ambitious.

I think for reliable results we shall have to revert to half a dozen short treatments spread over the space of a fortnight. A fortnight or even a month is of no consequence when treating such an inveterate disease as ringworm.

Alopecia areata of the parasitic variety, by which I mean both those cases which exhibit note-of-exclamation hairs at the borders of the patches, and those which present a black dotted, nutmeg-grater surface—often called black-dot ringworm—are curable by depilation brought about by *x*-rays, and my experience is that there is far less danger of dermatitis in these cases than in the ordinary small spored ringworm of childhood. Cases of sycosis, which so often resist all other treatment, can be safely attacked with *x*-rays, as even if a moderately severe dermatitis results, and permanent baldness is left, this is of little consequence in the beard region. Acne vulgaris can often be cured by *x*-rays when other means have failed; but it is only fair to mention that in one case where I had signally failed to bring about a cure Dr. Eyre was successful, using Wright's vaccine method against staphylomycosis.

Hypertrichosis is another disease in which *x*-rays may be safely and boldly exhibited—I say boldly for this reason: even if mild dermatitis occurs, the scar left is so slight that it can hardly be discerned, and therefore is infinitely preferable to the condition for which the treatment was applied, for apparently the only permanent result left is destruction of the hair follicles.

Fig. 13.—Before.

Fig. 14.—After.

Case of Warts, Removed by *x*-rays.

As I have said before, there are many cases of lupus and scrofulodermia, in the treatment of which *x*-rays are to be preferred to Finsen light. Ulcerated cases which cannot stand the pressure required by the latter treatment, lupus with much thickening and induration—these are the cases in which *x*-rays are indicated at all events at first.

As soon as *x*-rays have made the case amenable to Finsen light, the former should be discontinued, because a very prolonged treatment with *x*-rays, although curing the lupus, leaves a scar disfigured by telangiectases, which the Finsen scar avoids. Rodent ulcer is another disease in which *x*-rays have proved a most valuable remedy.

It cannot be said the *x*-rays are sure to cure every case of rodent ulcer, but it is well worth a trial in all cases, for the successful cases are, in my experience, quite 70 per cent. Recurrence is rare afterwards, and appears to be successfully met with further treatment.

Sequeira's experience bears this out. Latterly he has in all cases of *x*-ray treatment been prescribing treatments only once a week or fortnight, instead of three times weekly. I feel sure that, taking the cumulative action of the rays into account, this is a move in the right direction.

Lupus erythematosus is unlikely to benefit with *x*-rays; in fact, their application is more likely to be fraught with evil than with good.

It is perhaps among the rarer cases of skin disease that the most remarkable results have been obtained.

Thus, mycosis fungoides, one is justified in saying, is curable by means of *x*-rays. Whether the cure is rarely a permanent one is not yet certain, but in a case which was discharged cured nearly two years ago there has been no relapse; at least, I saw the patient after a year, when he was still quite well, and he was warned to return for further treatment at the slightest reappearance of the disease, and he has not come back.

In Kaposi's disease the *x*-rays have proved similarly successful.

Chronic ulcers, such as varicose and perforating, have been treated successfully, and *x*-rays may prove a valuable addition to our means of treating these inveterate cases.

With regard to malignant disease, whether sarcoma or carcinoma, the only case in which I have seen the tumours actually atrophy was one of sarcomatosis cutis in an old man, in which the disease was far too extensive to hope for a successful result. Nevertheless, some of the tumours treated did disappear.

I think that certainly the pain of carcinoma is relieved by *x*-rays, and I think also that some cases appear to be retarded in their growth. Multiple warts can be cured, and some of the blood diseases associated with enlarged spleen may be treated with benefit.

Before leaving the subject of *x*-rays I must just mention the cases of dermatitis which result from over-exposure.

Clinically I think these may be described as embracing two varieties—the acute and the chronic. The former may occur after one, two, or more over-exposures, and causes superficial ulceration, which heals somewhat slowly. The latter is far more insidious, and is met with as the cumulative action of the rays after many weeks or months. This is far more serious, and, when fully established, appears to be incurable, and tends to terminate in epithelioma. The acute variety is apt to cause permanent alopecia in cases of ringworm, while the chronic dermatitis must be carefully guarded against by all workers in *x*-rays.

In the hands, where it is naturally most often met with, the earliest symptoms are undue redness and a shiny appearance around the nails, which themselves appear to grow more quickly than normally, and hence require more frequent cutting.

---

### IV.—Radium Emanations and High Frequency Electricity.

As I have said before, treatment with radium has proved disappointing.

The salts used in therapeutics have been the bromide, the nitrate, and a compound radium and barium salt.

The marvellous physical properties of radium, discovered in 1903 just when the value of *x*-rays in therapeutics was becoming generally recognised, and aided by sensational articles in the lay papers, all conduced to first of all magnifying its value in disease, and *per contra* as is always the case when great expectations are disappointed, to its almost entire oblivion at the present time.

To be frank, generally speaking, as a therapeutic agent radium is not worth its cost; but it has in a decidedly limited field a certain value.

It was hoped, for instance, at one time that inhaling the emanations of radium might cure phthisis; this hope has not materialised.

Radium is useful in cases of rodent ulcer, when the disease is not extensive, and when for some season or other *x*-rays are not available.

Lupus of the mucous membrane of the nose or other small cavity, inaccessible to Finsen light or *x*-rays, is another indication for its use. I think also that, owing to its safety, radium is perhaps *the* agent of radiation for ophthalmic surgery, in the few cases where it may be desirable.

Owing to its expense, small quantities only are usually available, and therefore the treatments require to be prolonged, lasting 30 minutes or an hour.

But the sittings can be quite safely left for the patient to carry out.

The radium is, I think, best applied in a small sealed glass tube, in a holder made of some such material as copper wire, which can readily be made by anyone.

High frequency electricity is another form of radiation which experience has on the whole proved to be disappointing.

Personally, I strongly object to such terms as d'Arsonvalisation, effleuve, auto-condensation, etc.; they smack of quackery, and appear invented to dumbfound the multitude and to white-sepulchre the whole proceeding.

It is commonly said that high frequency electricity is practically the same as static electricity. Nothing from a physical standpoint could be further from the truth: the basis on which this statement rests is that the same class of patient receives equal benefit from both.

And the class of patient is the neurotic—I need say nothing further.

Provided that treatment by suggestion is not abused, and particularly in these days of nervous breakdowns, neurasthenia, and so forth, both high frequency and static electricity should prove very valuable additions to treatment.

Static electricity also seems capable of being useful in other fields; but this paper is endeavouring to deal with radiations and not electricity generally, so it would be out of place to discuss this further.

With regard to high frequency, it was at one time claimed that it was capable of causing sufficient anæsthesia for minor surgical operations; however, an investigation of the literature of the subject shows that this claim is an empty one.

Other diseases on which it has been tried are such widely different ones as diabetes, tuberculosis, keloid, alopecia areata, psoriasis, mucous colitis, and neurasthenia.

It will be noticed that these diseases have only one thing in common, and that is that they are often singularly rebellious to other forms of treatment.

I do not say that high frequency will eventually find no permanent place in therapeutics; but to be strictly honest, I must admit that in my hands this form of radiation has proved extremely disappointing, except in those cases where a functional neurosis was at the bottom of the trouble.

## Figures and Tables

**Fig. 9. Fig. 10. f1:**
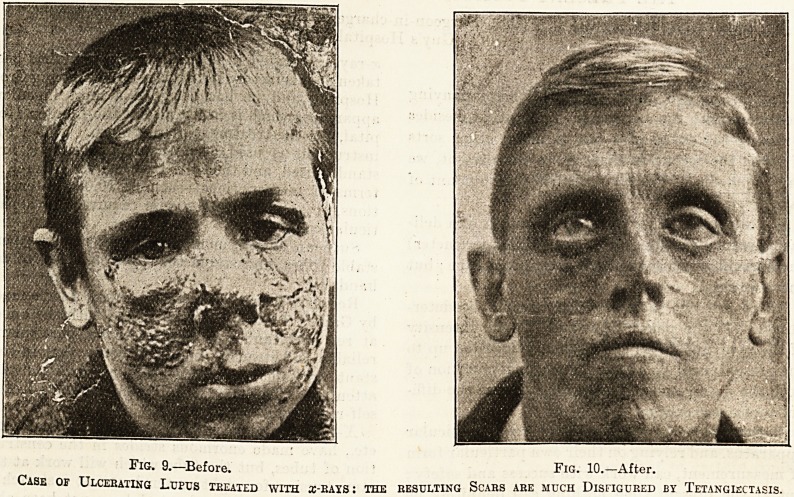


**Fig. 11. Fig. 12. f2:**
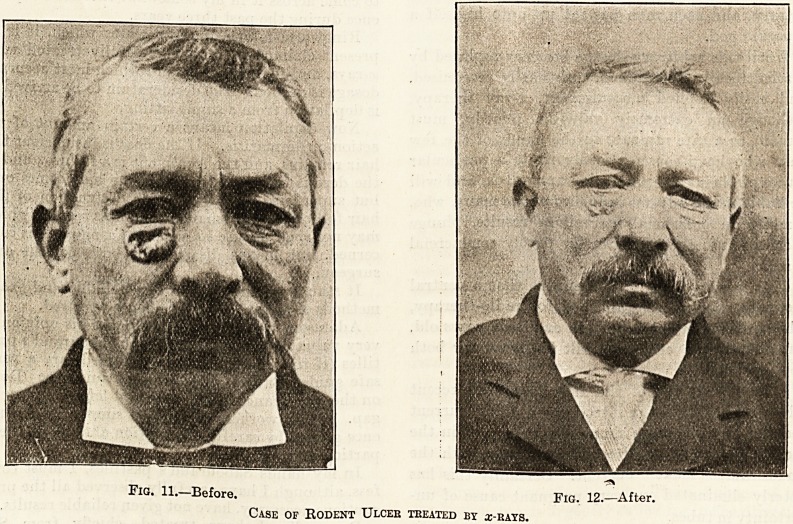


**Fig. 13. Fig. 14. f3:**